# Is it a policy crisis or it is a health crisis? The Egyptian context - Analysis of the Egyptian health policy for the H1N1 flu pandemic control

**DOI:** 10.11604/pamj.2013.14.59.1631

**Published:** 2013-02-12

**Authors:** Sameh Seef, Anders Jeppsson

**Affiliations:** 1 Department of public health, faculty of medicine, Lund University, Sweden

**Keywords:** Swine, flu, Egypt, health, policy, minority, corruption, epidemics, influenza

## Abstract

A new influenza virus that was first detected in people in April 2009, was initially referred to colloquially as “swine flu”, since it contained genes from swine, avian and human influenza viruses. It can, however, not be transmitted by eating pork or dealing with pigs. In Egypt, several hundred thousand pigs were killed in May, in spite of advice from global health authorities that such an action was unnecessary. Pigs are raised and consumed mainly by the Christian minority, which constitute some 10% of the population. Health Ministry estimated there were between 300,000-350,000 pigs in Egypt. This paper will analyze the Egyptian health policy for controlling the pandemic H1N1 flu, exploring its context, content, process, and actors. The analysis is based on the Leichter Context, which refers to systemic factors-political, economic and social, both national and international-that may have an effect on health policy, and is based on data collected from literature review and policy documents. The International health officials said the swine flu virus that has caused worldwide fear is not transmitted by pigs, and that pig slaughters do nothing to stop its spread. The WHO stopped using the term “swine flu” to avoid confusion. In Egypt, even the editor of a pro-government newspaper criticized the order to slaughter: “Killing (pigs) is not a solution, otherwise, we should kill the people, because the virus spreads through them,” wrote Abdullah Kamal of the daily Rose El-Youssef. The World Health organization also criticized the decision. The extinction of the Egyptian pigs is an example of how a health issue can be used to persecute a minority within a country. Although the current influenza has nothing whatsoever to do with pigs, the previous name of the epidemic was used as an argument to violate the rights of the Christian minority in Egypt.

## Essay

This paper will analyze the Egyptian health policy for controlling the pandemic H1N1 flu, exploring its context, content, process, and actors. The analysis is based on the Leichter Context, which refers to systemic factors-political, economic and social, both national and international-that may have an effect on health policy, and is based on data collected from literature review and policy documents [1].

A new influenza virus that was first detected in people in April 2009, was initially referred to colloquially as “swine flu”, since it contained genes from swine, avian and human influenza viruses. It can, however, not be transmitted by eating pork or dealing with pigs [2]. Globally the WHO uses a series of six phases of pandemic alert as a system for informing the world of the seriousness of the threat and of the need to launch progressively more intense preparedness activities. The world is presently in phase 6: a new influenza virus subtype is causing disease in humans, and spreading efficiently and sustainability among humans [3].

On July 16, 2009, the WHO the World Health Organization stopped producing global tables showing the numbers of confirmed cases for all countries. So the 2009 influenza pandemic has spread internationally with unprecedented speed. In the past pandemics, influenza viruses have needed more than six months to spread as widely as the new H1N1 virus has spread in less than six weeks [3]. In Egypt, several hundred thousand pigs were killed in May, in spite of advice from global health authorities that such an action was unnecessary. Pigs are raised and consumed mainly by the Christian minority, which constitute some 10% of the population. Health Ministry estimated there were between 300,000-350,000 pigs in Egypt [4].

Generally, in societies where formal hierarchies important it may be difficult to question or challenge high officials or elder statesmen. The position of ethnic minorities or linguistic differences may lead to a situation where certain groups being poorly informed about their rights, or services that do not meet their particular needs [5, 1].

Specifically, in Egypt under the present conditions and the activation of emergency law, power is mainly exercised by the government and top officials and expressed as thought control. In other words, power is a function of the ability to influence others by shaping their preferences.

The issue of the Egyptian pigs is an example of elite which can manipulate the values of the masses to reflect their own. Interest groups exist but they are not all equally powerful and do not have equal access to the policy making process. The values of the elite are conservative and consequently any policy change is likely to be incremental [6].

### Political systems: participation, benefits and openness

Egypt is constitutionally a democratic republic based on a multiparty system. The 1971 Constitution provides for the separation of powers between the Executive, Legislature and Judiciary. No constitutional changes have taken place in Egypt since 1980. In the 1980 referendum, the current president, Hosni Mubarak, assumed office with a two-thirds majority vote of the parliament, or People's Assembly (PA). (The president is currently in his fifth presidential term). He holds wide-ranging authorities and is the supreme commander of the armed forces, chair of the higher council for police agencies, and the higher council for judicial entities. The president nominates ministers, appoints 10 of the 454 members of the PA and 88 of the 264 members of the Shura Council, appoints and dismisses governors, university chairs, and other high ranking officials. The president is also the chair of the ruling National Democratic Party (NDP), which has been in power since it was established by former President Anwar Sadat in 1978 and effectively controls local government, the media, and the public sector [7].

Egypt's 16 legally registered opposition parties’ ability to compete has been frustrated by the NDP's dominance in the PA with a large majority of almost 90% (Figure 1). However, NDP has experienced a disappointing result in the parliamentary elections of 2000, which has prompted the substantial changes introduced to reform the party led by the President's son Gamal Mubarak [7].

In terms of openness of the political system the issue currently predominating public discussions is that of “banned” political groupings, i.e. Islamic groups, in particular the Muslim Brotherhood. Standing as independents during the 2000 parliamentary elections, and despite government efforts to discourage participation, the Brotherhood emerged with 17 seats-the largest opposition grouping. Extremist Islamists were responsible for the 1992-99 insurgency aimed at overthrowing the regime and instituting an Islamist state. Drawing followers from the younger and middle classes, the power base of the militants lies in the slums of Cairo and Upper Egypt where poverty and unemployment are widespread. But, weakened by internal divisions and a sustained campaign against militants by the state security forces, as well as a public backlash following the Luxor attack in 1997 where 58 tourists and 4 Egyptians were killed-the militants announced an unconditional ceasefire in March 1999, which is still in force [7].

The results of a household survey showed that the public has little trust in the representational mechanisms of the political system. The mainstream political culture in Egypt still considers public affairs to be governmental a. airs, under the sole authority of central administrators who decide without being accountable to public “demands”. Moreover, representative institutions are unable to mobilize public awareness and gain public trust. Hence, 97% of the household survey respondents do not attend political party meetings or rallies, whilst 94% of them do not discuss community problems with their local representatives, and 99% have never wrote to a newspaper to press for their interest [7].

Women's representation in the political system is marginal. The current People's Assembly includes only 11 women (2.43%). Youth participation is also a matter of interest at the national and local level. In late 2000, the President declared a new policy to encourage youth participation in public life [7].

Almost all political parties tend to be elitist, male-dominated and ageing entities, which are dominated by “historic” leaders (e.g. the Unionist, the Labour, and the Umma parties) causing internal splits between “younger” and older generations (e.g. the Nasserist and the Wafd parties). None of the parties explicitly exclude social groupings, for the law regulating political activity preconditions that all parties should be open to all Egyptians. However, most of the established parties are characterized by social classes, (e.g. the NDP being “the government” party, the Wafd representing the upper strata of professionals and liberal entrepreneurs, the Unionist party the radical leftists, and the adjourned Socialist Labour Party representing the Social Islamists) [7].

This paper analyzes the Egyptian health policy for controlling the pandemic H1N1 flu, exploring its context, content, process, and actors. The analysis is based on the Leichter Context, which refers to systemic factors-political, economic and social, both national and international-that may have an effect on health policy, and is based on data collected from literature review and policy documents.

### Problem identification and issue recognition

H1N1 has swept around the world in weeks, infecting millions and killing more than 4735 by official counts. It could worsen as temperatures cool in the Northern Hemisphere, making conditions better for viruses. H1N1 (also referred as “swine flu”) is a new influenza virus causing illness in people. This new virus was first detected in people in the United States in April 2009 [3]. As of 11 October 2009, worldwide there have been more than 399232 laboratory confirmed cases of pandemic influenza H1N1 2009 and over 4735 deaths reported to World health organization [3, 4].

The WHO Regional Office for the Eastern Mediterranean (EMRO) reported 13855 cases and 90 deaths. As of 10 October 2009, 23:00 hours, Cairo time, 13,855 laboratory-confirmed cases of Pandemic (H1N1) 2009 were reported to WHO by 21 out of 22 Member States of WHO Eastern Mediterranean Region. Djibouti became the latest country in the Region to report cases of pandemic (H1N1) 2009. There are 90 related deaths from Pandemic (H1N1) 2009 reported, so far, from 12 member states in the Region. These deaths were reported from Saudi Arabia (28), Oman (21), Yemen (11), Kuwait (9), Islamic Republic of Iran (7), Bahrain (4), Egypt (2), Lebanon (2), Syrian Arab Republic (2), Iraq (2), Palestine (1) and Qatar (1) [8]. With the increasing incidence rate and wide spreading of the H1N1 flu all over the world with its fatality the Egyptian government considered the issue as high political issue to be place in the political agenda for the taking the necessary measure.

The pandemic flu was perceived as a crisis which opens the window for policy, where the problem stream, the policy stream and the political stream all are met together. According to Kingdon's model, the three streams work along different, largely independent channels until at particular times, which become policy windows, they flow together, or intersect. This is when new issues get onto the agenda and policy is highly likely to change [9].

### Policy formulation

According to the position map (Egypt flu policy formulation) (Table 1) most power was on the side of the government and policy makers and the majority of the Egyptian society presented by Muslims, whereas low power was presented by the poor farmers and pork industry represented by almost half million Egyptians, most of them are from the Coptic minority of the Egyptian population, where the church, WHO and NGOs reflected medium power but with no effect.

**Table 1:** Position map (Egypt flu policy formulation)

High opposition	Medium opposition	Low opposition	Neutral	Low support	Medium support	High support
Poor farmers	WHO	Church				Government
Pork industry	NGOs					President
						MOHP
						Media
						Muslims
Low power	Medium power					High power

On 4th of March 2009 the Egyptian parliament debated passing a law to prohibit raising of pigs and dealing with its products in all parts of Egypt. It was decided to send the bill to the concerned authorities for discussion. At the 27^th^ of April the minister of health and population presented the plan for addressing the flu pandemic and presented a plan to all the concerned ministries, including the ministries of education, transportation, environment, and agriculture. During the 27^th^ session parliament members mentioned that “Egypt is an Islamic country and it is logically to “requesting killing all the pigs in the country, with compensating the farmers for that.”

### Policy implementation

At the 28^th^ of the same months after days of the previous discussion, the Egyptian parliament decided to pass the bill concerning the killing all the pigs in Egypt, to be carried out as soon as possible. At the 30^th^ of April 2009 Egypt began slaughtering the roughly 300,000 pigs in the country as a precaution against swine flu even though were no cases had been reported there. At the 17^th^ of May 2009 the parliament debated an urgent request from some members about compensating the farmers in satisfactory ways, after they had been badly treated by the officials and the way their pigs had been slaughtered.

The policy implementation had been carried out through top-down approach where the entire policy process passed as a linear sequence of activities in which there was a clear division between policy formulation and policy execution. Goals had been clearly defined and widely understood, the necessary political, administrative, technical and financial resources were available, a chain of command had been established from the centre to the periphery, and a communication and control system had been in place to keep the whole system on course but pig farmers - overwhelmingly Christian - were angered. Government efforts to start the slaughter Wednesday were met with farmers who hurled stones at Health Ministry trucks.

### Policy evaluation

When evaluating the Egyptian health policy for the control of the swine flu pandemic, we need to look at some important aspects and look carefully in the process of making and implementing the policy which mainly discussed and carried out in few days even were no cases reported in Egypt, affecting many poor Coptic Egyptian families mainly living of the little income coming from raising the pigs.

Egypt′s government was hoping to look strong and proactive in the swine flu scare with its decision to slaughter all the country′s pigs, after having undergone heavy criticism at home for poor planning and corruption.

Egypt, which has no swine flu cases, is the only country in the world to order a mass pig slaughter in response to the disease. The move mirrored Egypt′s battle with bird flu, in which the government killed 25 million birds within weeks in 2006.

But international health officials said the swine flu virus that has caused worldwide fear is not transmitted by pigs, and that pig slaughters do nothing to stop its spread. The WHO stopped using the term “swine flu” to avoid confusion. In Egypt, even the editor of a pro-government newspaper criticized the order to slaughter: “Killing (pigs) is not a solution, otherwise, we should kill the people, because the virus spreads through them,” wrote Abdullah Kamal of the daily Rose El-Youssef [10]. The World Health organization also criticized the decision.

## Conclusion

The extinction of the Egyptian pigs is an example of how a health issue can be used to persecute a minority within a country. Although the current influenza has nothing whatsoever to do with pigs, the previous name of the epidemic was used as an argument to violate the rights of the Christian minority in Egypt.
